# Simultaneous Detection of B-Cell Chronic Lymphocytic Leukemia and Colon Adenocarcinoma in the Same Mesenteric Lymph Node

**DOI:** 10.4274/tjh.2012.0169

**Published:** 2013-03-05

**Authors:** Ahmet Emre Eşkazan, Selin Berk, Ferhat Özden, Sibel Erdamar, Nükhet Tüzüner, Teoman Soysal

**Affiliations:** 1 Diyarbakır Training and Research Hospital, Department of Hematology, Diyarbakır, Turkey; 2 İstanbul University Cerrahpaşa Faculty of Medicine, Department of Internal Medicine, İstanbul, Turkey; 3 İstanbul University Cerrahpaşa Faculty of Medicine, Department of Pathology, İstanbul, Turkey; 4 İstanbul University Cerrahpaşa Faculty of Medicine, Department of Internal Medicine, Division of Hematology, İstanbul, Turkey

**Keywords:** Chronic lymphocytic leukemia, CLL, Colon adenocarcinoma

A 50-year-old male patient presented with microcytic anemia (hemoglobin: 10.2 g/dL, hematocrit: 33.8%, mean corpuscular volume: 64 fL), leukocytosis of 19.7 x 109/L (lymphocytes of 51.2%, 10x109/L), and normal platelet count. During the etiological work-up of his anemia an abdominal computed tomography (CT) scan was done, which showed a mass of 6.5x5.5 cm located in the ascending colon and hepatic flexura causing a wall thickening of 20 mm. The thoracic CT was normal. A complete colonoscopy showed an ulcerovegetative lesion in the transverse colon narrowing the lumen and multiple biopsies were performed, which revealed a well-differentiated colon adenocarcinoma. During the surgical removal of the tumor, 4 peripancreatic and 48 mesenteric lymph nodes and the perilymphatic fat tissue were resected, which all had diffuse infiltration of atypical lymphocytes. In one mesenteric lymph node, both invasion of the colon adenocarcinoma and atypical lymphocytes were demonstrated (Figures 1A and 1B). The atypical lymphocytes were immunohistochemically positive for CD20 (Figure 2), Bcl-2, CD23, and CD5 consistent with B-cell chronic lymphocytic leukemia/small lymphocytic lymphoma (B-CLL/SLL). After the patient was referred to the hematology department, flow cytometry was performed from the peripheral blood, which was also consistent with B-CLL/SLL. He had early-stage CLL and so we decided to monitor the disease; the patient was referred to the medical oncology department for the treatment of colon adenocarcinoma. 

Patients with CLL have more than twice the risk of developing a second cancer, and this increased incidence is attributed to disease- or therapy-related immunosuppression [1]. The most common types of cancers developing in CLL patients are skin cancers, soft-tissue sarcoma, colorectal and lung carcinoma [2]. The incidental detection of CLL/SLL based on the histological evaluation of the lymph nodes resected for rectal adenocarcinoma is a rare entity [3,4]. 

The synchronous diagnosis of B-CLL/SLL and colon adenocarcinoma in our case is most probably coincidental. However in the synchronous presentation of these two malignancies, an epidemiological association has been noted [5], and this synchronous relationship can also be explained in terms of the immunosuppression over a prolonged period of time. 

**Conflict of Interest Statement**

All authors have no conflict of interest to declare.

## Figures and Tables

**Figure 1:**
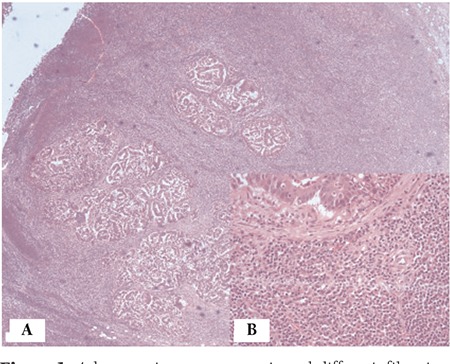
Adenocarcinoma metastasis and diffuse infiltration of small lymphocytes in the same mesenteric lymph node (A: hematoxylin and eosin, 40x; B: hematoxylin and eosin, 400x).

**Figure 2:**
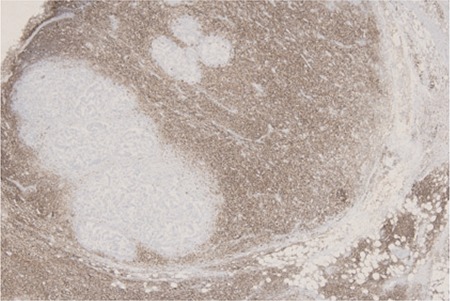
CD20 is positive in diffuse lymphoid infiltration, whereas it is negative in metastatic glands (40x).
